# Type III monteggia injury with ipsilateral type II Salter Harris injury of the distal radius and ulna in a child: a case report

**DOI:** 10.1186/1756-0500-7-156

**Published:** 2014-03-17

**Authors:** Huw LM Williams, Thayur R Madhusudhan, Amit Sinha

**Affiliations:** 1 Trauma and Orthopaedic Department, Glan Clwyd Hospital, Sarn Lane, Bodelwyddan, Rhyl LL18 5UJ, UK

**Keywords:** Monteggia fracture, Clinical and radiological examination, Surgery

## Abstract

**Background:**

Although previously reported, ipsilateral Monteggia fracture dislocation and distal radius fracture in a child is still a rare occurrence. A full clinical examination may be difficult but should not be ignored. Full length forearm radiographs are ideal but proper limb positioning may be difficult. The injury pattern can be easily missed.

**Case presentation:**

A five-year- old right hand dominant Caucasian male presented with a history of fall on outstretched hand. Clinical examination was difficult and X - rays confirmed type III Monteggia fracture with an ipsilateral Type II Salter Harris injury of the distal radius and ulna.

**Conclusion:**

This report highlights the need for relevant examination of the wrist and elbow in young children. Appropriate radiographs must also be performed to prevent missing these injuries.

## Background

Monteggia fracture patterns are rare in children and merits appropriate treatment to avoid late disability of the elbow and forearm. The injury pattern is easily missed initially or later if not followed up adequately. Both operative and non operative treatment methods are described and are equally successful. The chosen method will be dictated by the injury pattern, associated skeletal injuries and the stability of reduction achieved intra-operatively. We report this rare injury with associated distal radius and ulna injury in the ipsilateral limb, the importance of a full thorough clinical examination and the need for full length radiographs in order not to miss this injury particularly by the junior doctors in the emergency department.

## Case presentation

**Figure 1:**
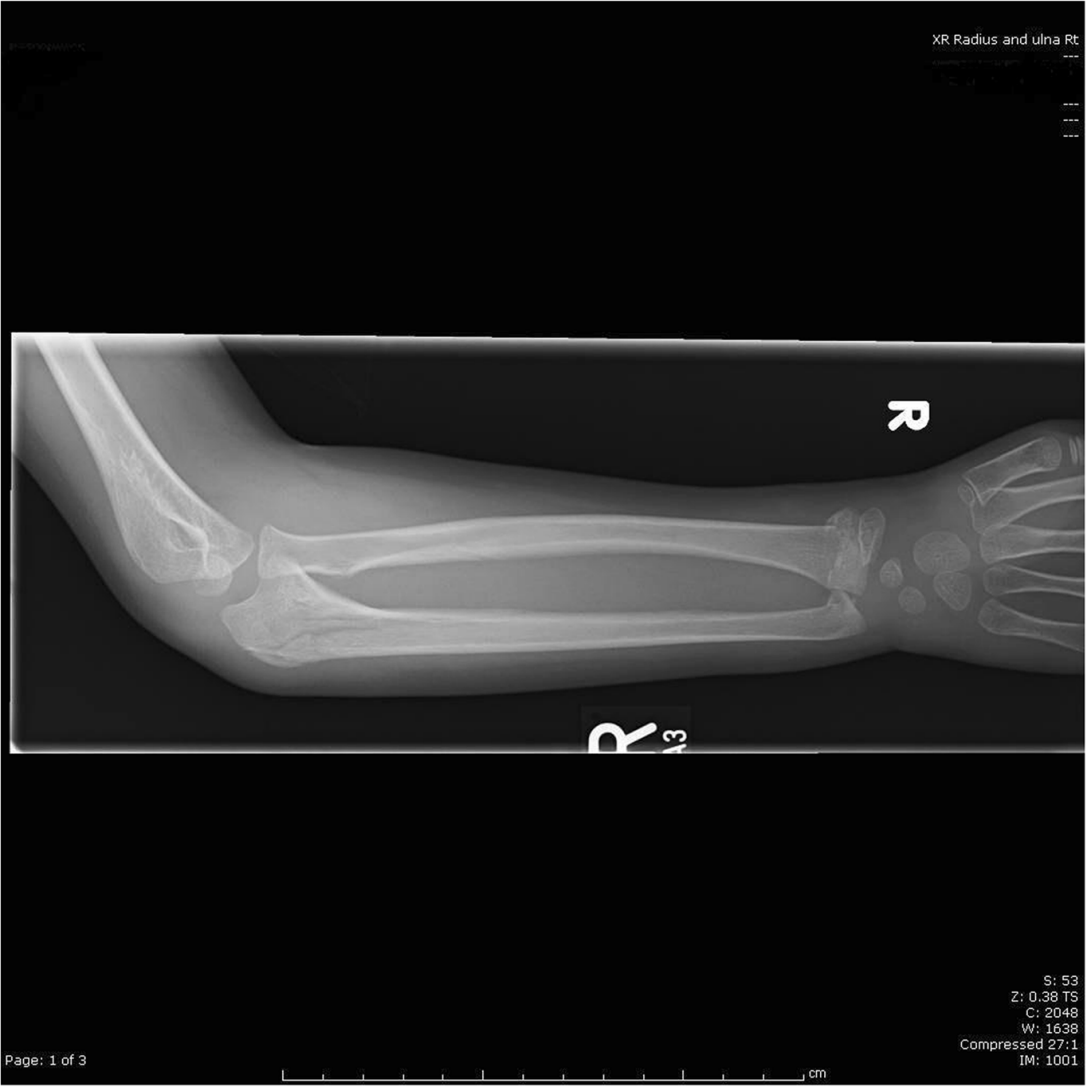
Full length anteroposterior X ray of the forearm.

**Figure 2:**
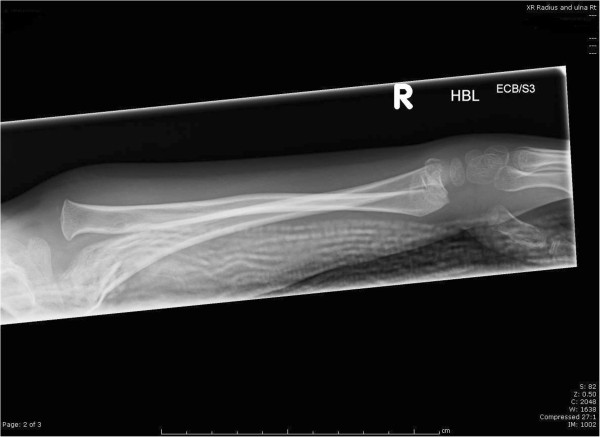
Full length lateral X ray of the forearm.

A five-year- old, right hand dominant, Caucasian male, fell from a height of five feet onto his right out stretched hand. He noticed pain and swelling around his wrist as a result and presented to the accident and emergency department three hours post injury. Upon examination, he had a deformity of his dominant right wrist and forearm, with tenderness and swelling of the right distal wrist. The forearm was mildly swollen and tender over the volar aspect all along the length. There were no signs of compartment syndrome. Forearm and elbow movements were painful and restricted. There were no distal neurovascular deficits. There were no associated skeletal injuries. Full length forearm radiographs were requested in keeping with the clinical picture which confirmed a Type II Salter Harris injury to the distal radius and ulna. There was an angulated fracture of the ulna associated with anterolateral dislocation of the radial head (Figures 1, 2). The limb was supported with a plaster slab and listed for emergency theatre.

**Figure 3:**
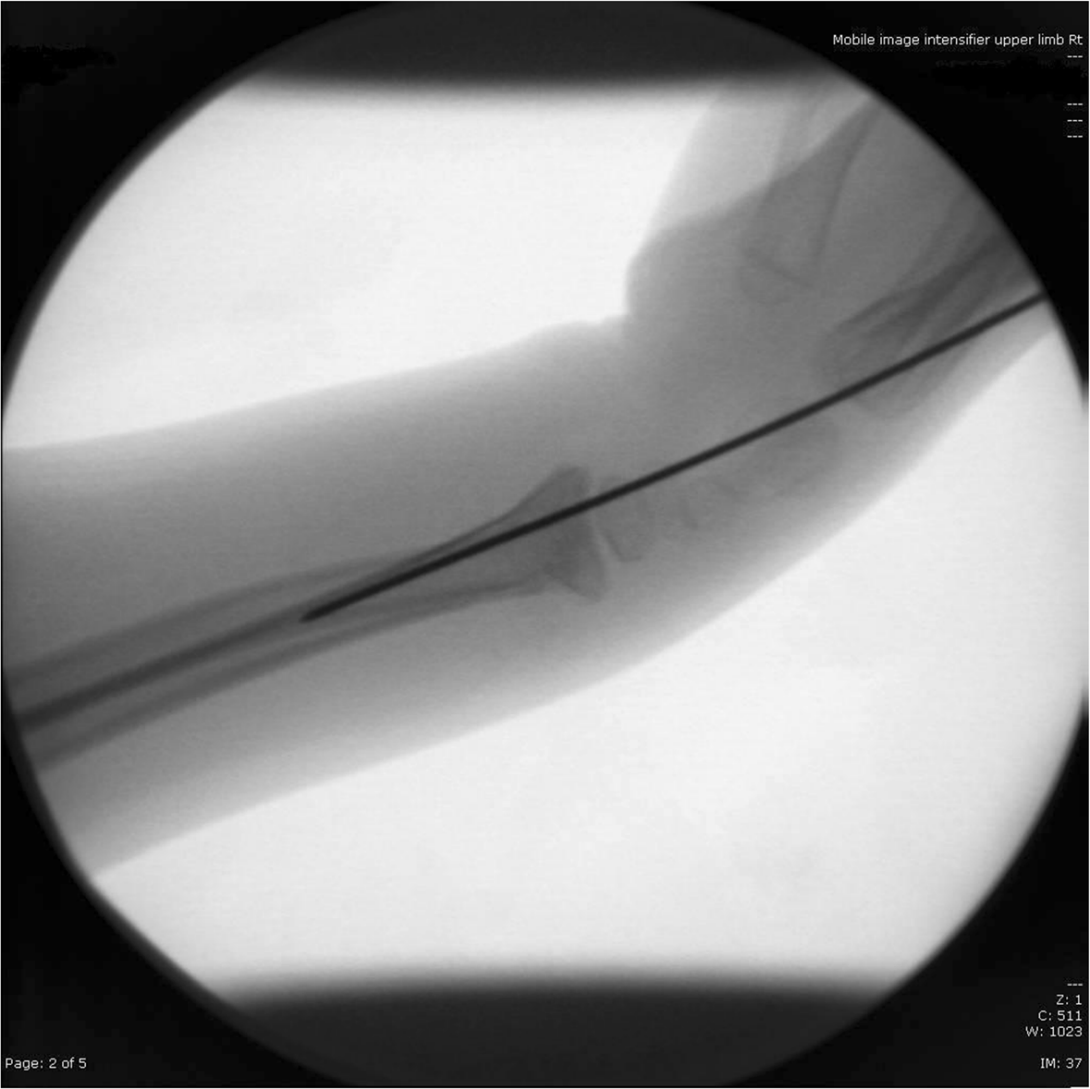
Intraoperative lateral X ray of the wrist.

**Figure 4:**
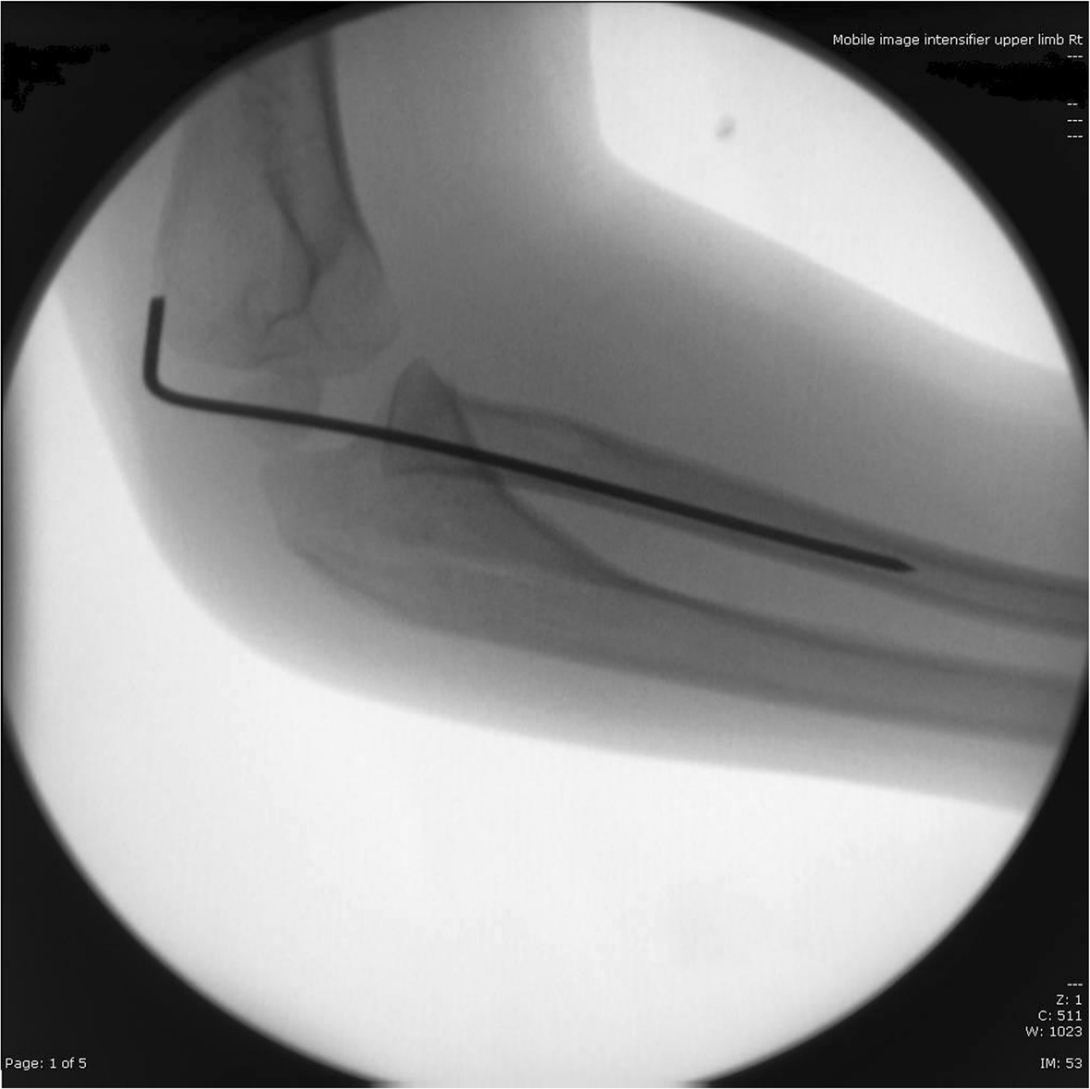
Intraoperative lateral X ray of the elbow.

**Figure 5:**
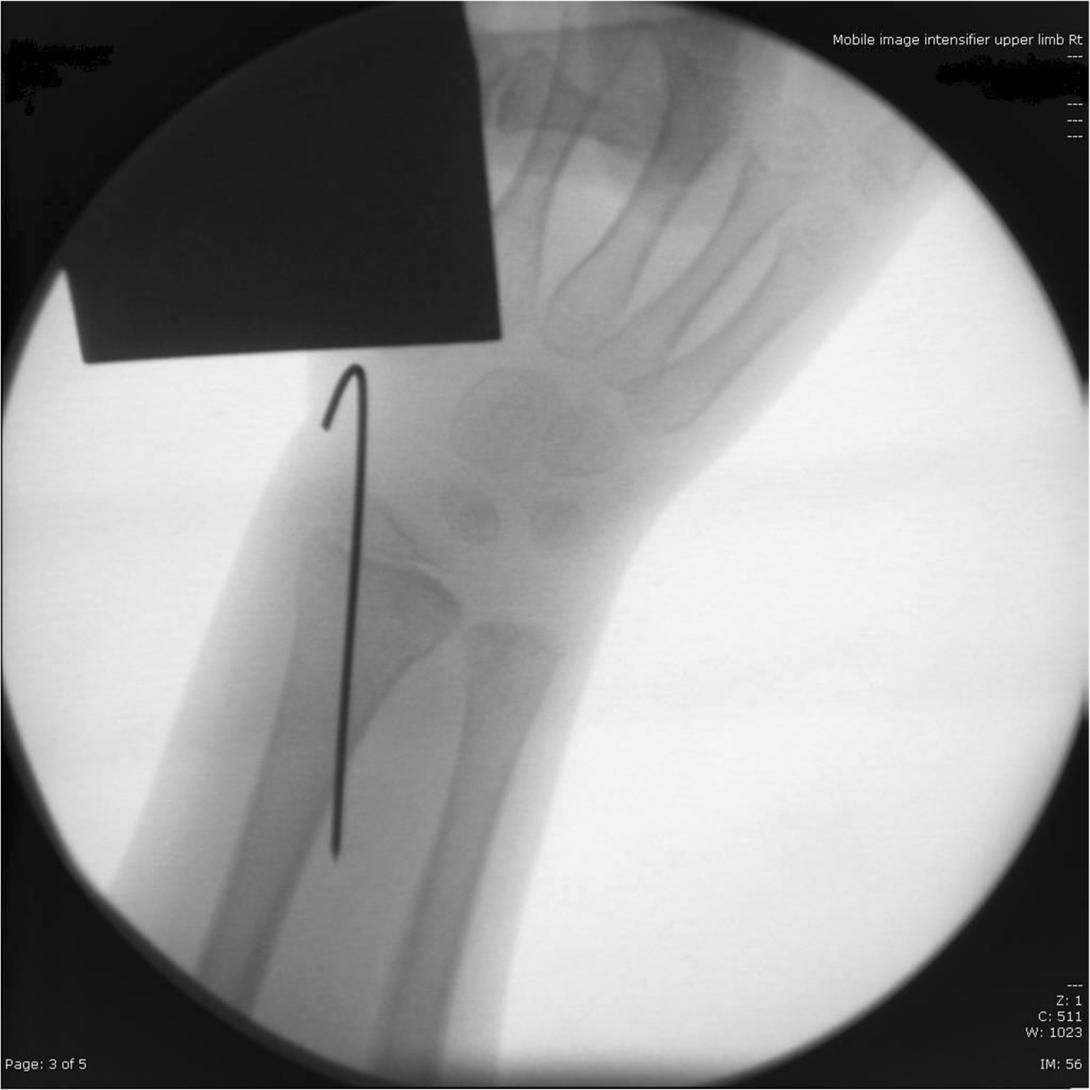
Intraoperative antero-posterior X ray of the wrist.

The patient was anaesthetised and positioned supine. The distal radius fracture was manipulated under anaesthesia and stabilised with k wire. The radial head was then relocated by closed method and ensuring adequate reduction in full supination, stabilised with a radio- capitellar wire as the radial head relocation was unstable. (Figures 3, 4, 5). The ulna fracture was well aligned and therefore was treated non-operatively. The limb was immobilised in an above elbow resting plaster in 90 degrees elbow flexion and in full supination of the forearm.

**Figure 6:**
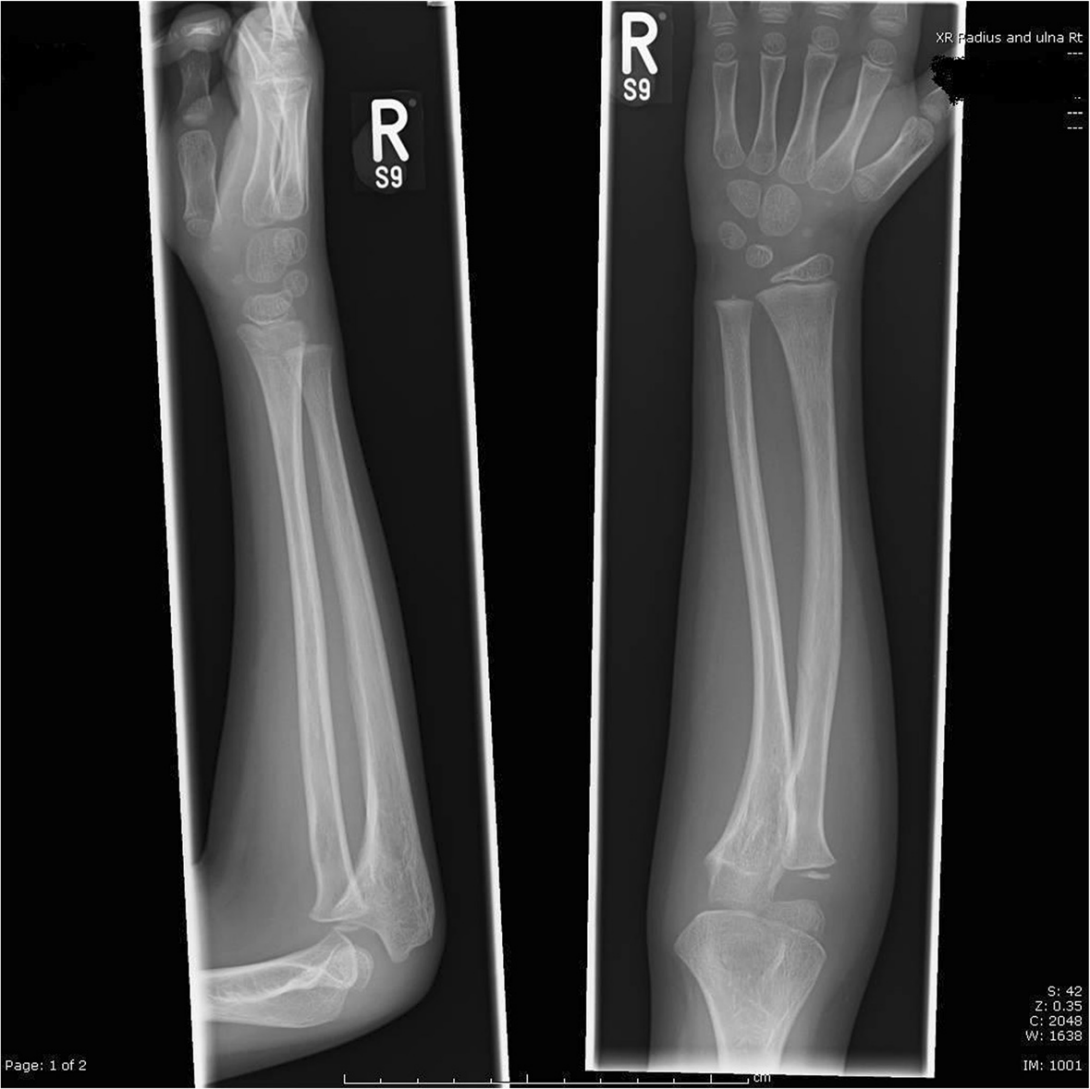
6 month follow up antero-posterior and lateral X rays of the forearm.

**Figure 7:**
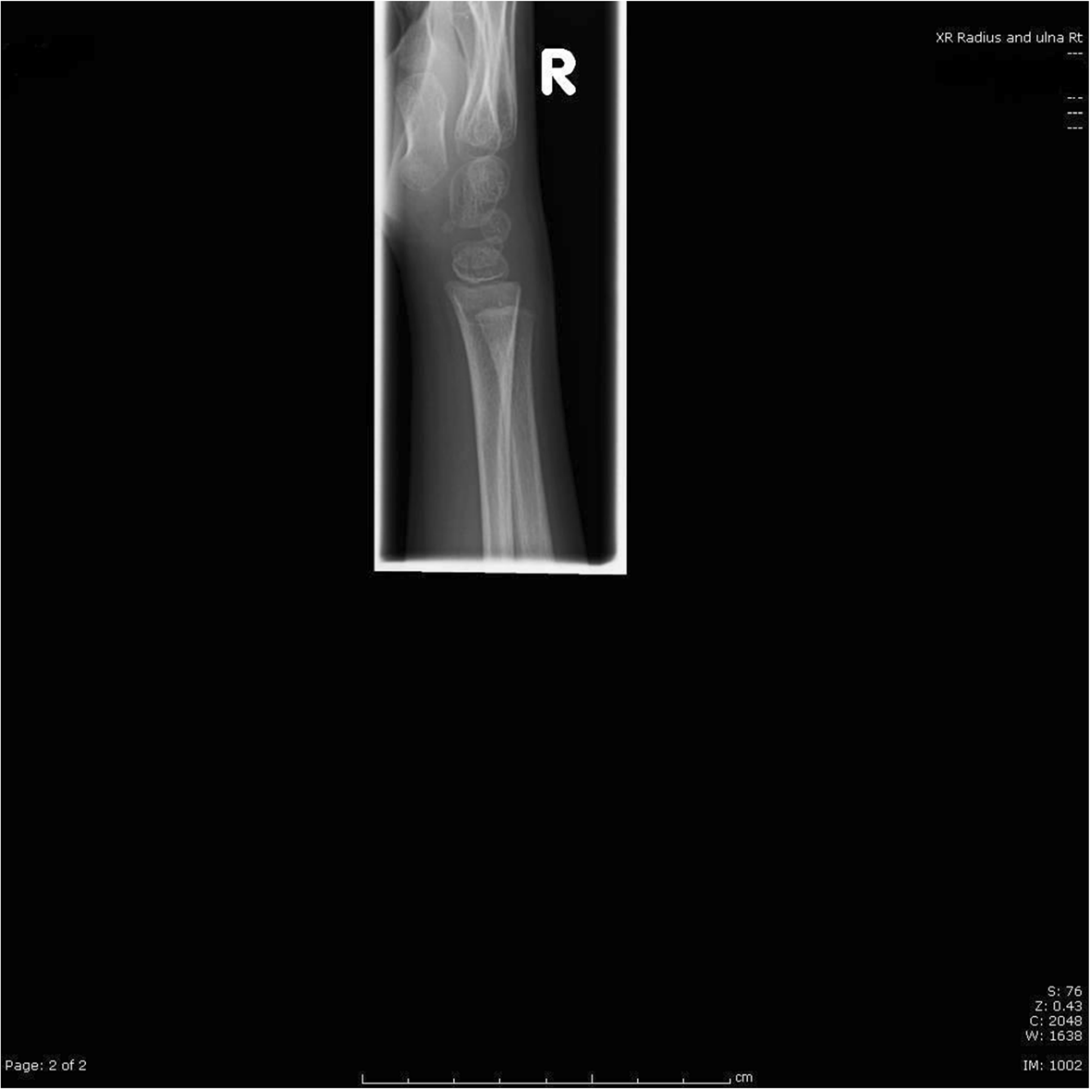
6 month follow up lateral X ray of the wrist.

The patient was monitored for compartment syndrome over the next 24 hrs and was discharged when clinically safe. He was followed up in clinic weekly for 3 weeks, with serial radiographs of the forearm. The wires were removed after 5 weeks under anaesthesia. The elbow and forearm were initially stiff and was referred to physiotherapy for gentle elbow, forearm and wrist mobilisation. At 2 months follow up the patient had regained full flexion, extension and pronation. Supination was reduced in the final 30 degrees. At 6 months there was 10 degrees hyper-extension at the elbow with full flexion, full forearm rotations, full range of movement at the wrist and he had full functional use of his hand. Follow up radiological images showed all fractures had healed (Figures 6 and 7).

## Discussion

Giovanni Monteggia, based on cadaveric studies, described the pattern of injury in adults but the peak incidence occurs in the age range of 4–10 and represents 0.4% of all forearm fractures [1]. Due to the infrequent exposure of this type of injury it can be easily missed if not specifically looked into.

Bado later classified the injury into 4 subtypes depending on the direction of radial head dislocation [2]. Several variants have been further described particularly in children [3]. Of these injuries, type 1 (59%) and type-III (26%) are the most common [4,5]. Our patient sustained a type-III injury with an ipsilateral Type II Salter Harris distal radius and ulna fracture.

It has been estimated that, up to 50% senior house officers in accident and emergency departments and 25% of senior radiologists missed a Monteggia injury [6]. Our patient had a painful forearm coupled with restricted elbow and forearm movements, which heightened the suspicion. A good clinical examination of the elbow and forearm is therefore important to rule out this pattern of injury. This may be difficult in an uncooperative child but should be routinely practised. Appropriate full length radiographs are requested when clinical suspicion is high. This we feel goes a long way in identifying the injury and preventing late complications.

Non operative methods of reduction have been reported with successful outcomes [1,7-11]. These fractures - whether it be plastic deformation or incomplete fractures – tend to be stable and thus maintain the anatomical reduction in a cast achieving good results [1,5,7-11].

Operative intervention should be performed for failed closed reduction and in unstable fracture dislocation patterns with excellent results being achieved [6,11-15]. In our patient, the radial head was unstable after closed reduction and therefore the option of transcapitellar wiring was contemplated. We feel the instability pattern is more pronounced if there is an ipsilateral radius fracture, necessating operative stabilisation.

Percutaneous radial head pinning ensures maintenance of superior radio-ulnar articulation. Though concerns have been raised about the possibility of capitellar damage and subsequent physeal damage, we did not notice this in our limited period of follow up. However this is a possibility and only a prolonged follow up till skeletal maturity will confirm this occurrence. It is therefore important to communicate this to the parents in the consent process. The ulna fracture was aligned well and therefore it was decided to treat the same in a well moulded plaster cast.

## Conclusion

Unstable Monteggia fracture dislocation with ipsilateral distal radius fracture although rare is possible in children. The injury being uncommon can be easily missed particularly by junior doctors in the emergency department. The physician should have a low threshold for suspecting these types of injuries when examining children. A good clinical examination and full length radiographs of the forearm are mandatory in all suspected cases.

## Consent

Written informed consent was obtained from the patient’s legal guardian(s) for publication of this case report and any accompanying images. A copy of the written consent is available for review by the Editor-in-Chief of this journal.

## Competing interests

The authors declare that they have no competing interests.

## Authors’ contributions

HLW performed the literature review and contributed to the writing of the manuscript. TM was a major contributor in writing the manuscript and performed the operation. AS assessed the child clinically post-operatively and in follow up clinic. All authors read and approved the final manuscript.
